# Novel Paired Normal Prostate and Prostate Cancer Model Cell Systems Derived from African American Patients

**DOI:** 10.1158/2767-9764.CRC-22-0203

**Published:** 2022-12-13

**Authors:** Mira Jung, Keith Kowalczyk, Ryan Hankins, Gaurav Bandi, Bhaskar Kallakury, Michael A. Carrasquilla, Partha P. Banerjee, Scott Grindrod, Anatoly Dritschilo

**Affiliations:** 1 Department of Radiation Medicine, Georgetown University School of Medicine, Washington, District of Columbia.; 2 Department of Biochemistry and Molecular & Cellular Biology, Georgetown University School of Medicine, Washington, District of Columbia.; 3 Department of Urology, Georgetown University School of Medicine, Washington, District of Columbia.; 4 Department of Pathology, Georgetown University School of Medicine, Washington, District of Columbia.; 5 MedStar Georgetown University Hospital, Washington, District of Columbia.; 6 Shuttle Pharmaceuticals, Inc, Rockville, Maryland.

## Abstract

Prostate cancer is the most frequently diagnosed solid malignancy in men. African American (AA) men are at greater risk for developing prostate cancer, and experience higher mortality rates, as compared with Caucasian American men. However, mechanistic studies to understand this health disparity have been limited by the lack of relevant *in vitro* and *in vivo* models. There is an urgent need for preclinical cellular models to investigate molecular mechanisms underlying prostate cancer in AA men. We collected clinical specimens from radical prostatectomies of AA patients and established 10 paired tumor-derived and normal epithelial cell cultures from the same donors, which were further cultivated to extend the growth under “conditional reprogramming.” Clinical and cellular annotations characterized these model cells as intermediate risk and predominantly diploid. Immunocytochemical analyses demonstrated variable expression levels of luminal (CK8) and basal (CK5, p63) markers in both normal and tumor cells. However, expression levels of TOPK, c-MYC, and N-MYC were markedly increased only in tumor cells. To determine cell utility for drug testing, we examined viability of cells following exposure to the antiandrogen (bicalutamide) and two PARP inhibitors (olaparib and niraparib) and observed decreased viability of tumor-derived cells as compared with viability of normal prostate-derived cells.

**Significance:**

Cells derived from prostatectomies of AA patients conferred a bimodal cellular phenotype, recapitulating clinical prostate cellular complexity in this model cell system. Comparisons of viability responses of tumor derived to normal epithelial cells offer the potential for screening therapeutic drugs. Therefore, these paired prostate epithelial cell cultures provide an *in vitro* model system suitable for studies of molecular mechanisms in health disparities.

## Introduction

Prostate cancer is the most frequently diagnosed solid malignancy in American men with an estimated 268,490 new cases of prostate cancer and 34,500 deaths in 2022 (ACS Facts & figutes 2022; ref. 1). Health disparity studies have shown a higher risk of developing prostate cancer as well as higher cancer-specific death rates in African American (AA) men as compared with Caucasian American (CA) men (2–4). Such health disparities have been attributed to differences in socioeconomic status, access limitations to health care services and delay of cancer diagnosis, environmental exposures and highly heterogenetic differences as potential causative factors (5). Studies performed at centers offering equal access for patients and adjusting for socioeconomic and lifestyle factors, have also reported differences in survival attributable to tumor biology (5, 6). In a study of 35 clinical trials, 1,843 patients with prostate cancer demonstrated survival differences by race (5). Such observations offer support for the possibility that genetically based factors in the biology of prostate cancers in AA patients may underlie the observed health disparities.

Previous studies have identified differences in gene expression in tumor biopsy specimens from AA men as compared with CA men (7–9). Microarray analyses of 69 clinically matched patients with prostate cancer demonstrated differences in gene expression profiles of prostate tumors from AA and CA men, particularly in genes related to tumor immunobiology (9). Our previous report of gene expression in prostate epithelial cells from AA and CA men show differences in the expression of genes affecting tumor aggressiveness and metastases using high-throughput microarray assays (8). These observations support primary epithelial cultures as a useful tool for discovering molecular mechanisms underlying health disparities in prostate cancer; however, the cultures permitted <5 passages, followed by cell senescence and cell death. Although this model permitted investigation of low-passage cellular events, including differential gene expression, further molecular and cellular characterization and testing of responses to drug therapy are poorly supported.

The development of “conditional reprogramming” (CR) technology indefinitely extends the life span of primary human epithelial cells using both mouse fibroblast feeder cells (J2) and the Rho-associated kinase (ROCK) inhibitor, Y-27632 (10). Cells under CR condition (CRC) have been evaluated for potential in personalized medicine applications by expanding the growth of a paired tumor and normal epithelial prostate cell cultures established from a prostatectomy specimen of a CA patient with organ confined Gleason grade 3+4 = 7 prostate cancer. Studies have shown that conditionally reprogrammed cells retain cell lineage commitment and maintain the heterogeneity of cells present in a biopsy (11–16). We reported these epithelial cell cultures; GUMC29 (“normal” prostate tissue derived) and GUMC30 (prostate “tumor” derived) as a novel “paired” set showing feasibility for comparative studies.

Here, we report the establishment of AA patient-derived prostate cell models for use in prostate cancer health disparities research. These prostate epithelial cells were derived from tumor and normal tissues obtained from AA men and offer a resource for cellular and molecular studies to enhance the understanding of mechanisms underlying carcinogenesis, cancer aggressiveness and resistance to therapy. Furthermore, comparisons of viability responses of tumor-derived with normal-derived cells offer the potential for precision medicine.

## Materials and Methods

### Patients with Prostate Cancer

Patients undergoing elective prostatectomies at the MedStar Georgetown University Hospital were enrolled into the tissue collection protocol under the Georgetown University's Institutional Review Board approval (IRB Study Number: 2017-0614). Written and signed informed consent was obtained from all patients, and the studies were conducted in accordance with recognized ethical guidelines as described in the Nuremberg Code, Declaration of Helsinki, and the Belmont Report. The Georgetown-MedStar IRB approval was received (2017–0614) and the trial was conducted in accordance with Good Clinical Practice guidelines. Fresh clinical specimens were transferred from the operating room to surgical pathology, processed and examined by the pathologist for areas of tumor or normal (non-tumor) regions for biopsies. The specimens were placed on ice and transferred to the laboratory for processing, outgrowth and expansion.

### Establishing Paired Prostate Cancer and Normal Epithelial Cells

Following gross inspection by an experienced pathologist, fresh tissues were dissected to generate primary cell cultures as described previously (17). The minced tissues were cultured in collagen-coated cell culture dishes with keratinocyte serum-free medium (KSFM) supplemented with human recombinant EGF and bovine pituitary extract at the time of use. Tissue explants were grown to confluence, and aliquots of the primary cultures were frozen and stored in liquid nitrogen until the cells were reestablished in secondary cultures. Cells from 58 patients were expanded up to 1–4 passages for banking. The nomenclature for labeling cells collected from 58 consecutive prostatectomy specimens include AP, CP, and AS for AA, Caucasian, and Asian, respectively. The number indicates the sequential clinical specimen and tumor (T) or normal (N) prostate tissue. The detailed protocol is summarized in Supplementary Protocol S1.

### Conditional Reprogramming Cell Culture (CRC)

To further expand primary cells, irradiated 3T3 J-2 cells derived from mouse embryonic fibroblast cells were used as feeder cells for growing epithelial cells. J2 cells were cultured in complete DMEM, 10% heat-inactivated FBS and 1% Pen Strep. The complete DMEM and Ham's F-12 nutrient mix (25%) supplemented with hydrocortisone, EGF, bovine insulin, cholera toxin, amphotericin B, gentamicin, and Rock Inhibitor (Y-27632) was used to grow primary epithelial cells with irradiated J2 cells with 30 Gy of gamma radiation (18). *Mycoplasma* detection assays were routinely performed using the VenorGeM qEP kit (Minerva Biolabs). The latest date for *Mycoplasma* detection of all cell cultures was on July 24, 2022. The detailed protocol for CRC is summarized in Supplementary Protocol S1.

### Cellular Authentication Analysis

Short tandem repeat (STR) DNA fingerprinting was performed to verify cell novelty. The process of obtaining a DNA profile using highly polymorphic STR repeats includes the isolation of DNA from cells, amplification of targeted areas of that DNA, and analysis of the amplified product using a genetic analyzer. STR DNA typing generates reproducible data in a format suitable for reference cell databases or patient sample comparisons. The procedures for cell authentication complied with the guidelines set forth by the ATCC SDO Workgroup ASN-0002 Standards document (19).

### Karyotype Analysis

Primary cells at 0–2 passages were cultured in medium containing no serum and using 0.05% trypsin to passage. Cells were subcultured onto 24 × 24 mm glass coverslips until optimal confluence was reached to maximize the number of metaphase cells. In general, 20 metaphase cells were analyzed. The methodology utilized in this analysis does not detect subtle rearrangements or low-level mosaicism and cannot detect microdeletions. Also, it cannot detect molecular cytogenetic abnormalities (such as microdeletions and microduplications) that may be detectable by microarray analysis.

### Cellular Microarray Analysis

Paired tumor and/or normal cell-based microarrays were prepared for studies employing immunostaining protocols. Analogous to conventional tissue microarray production, formalin-fixed paraffin-embedded cell-based microarrays were constructed using 5 × 10^6^ cells grown under CRC conditions. The cell pellet is then embedded into paraffin, processed and sectioned (5-μm) and arranged on slides. Slides are processed using standard immunostaining protocols. Briefly, slides were treated with 3% hydrogen peroxide and 10% normal goat serum for 10 minutes each and exposed to primary antibodies for P63 (1/200, Cell Signaling Technology, catalog no. 39692, RRID:AB 2799159), androgen receptor (AR; 1/50, Abcam, catalog no. ab108341, RRID:AB_10865716), and CK8 (1/800, Abcam, catalog no. ab59400, RRID:AB_942041) for 1 hour at room temperature. Slides were then exposed to either an anti-rabbit (Agilent, catalog no. K4003, RRID:AB_2630375) or anti-mouse (Agilent, catalog no. K4001, RRID:AB_2827819) horseradish peroxidase–labeled polymer from Agilent according to manufacturer's instructions. Slides were subsequently incubated with 3, 3′-diaminobenzidine tetrahydrochloride (DAB) chromagen (Dako) and then counterstained with hematoxylin (Thermo Fisher Scientific, Harris modified hematoxylin). Consecutive sections with the primary antibody omitted were used as negative controls. Alternatively, 8-well slides are plated directly with cells from normal and tumor pairs expanded using CRC. This approach requires relatively smaller numbers of cells (∼5 × 10^3^ cells).

### Genome Sequencing and Profiling

Genomic DNA was isolated from 5 × 10^6^ cells using the DNA Purification Kit (Promega). Whole-genome sequencing (WGS) was performed and analyzed at the Yale Center for Genome Analysis (West Haven, CT). Using Illumina's Sequence Analysis Viewer, various quality control (QC) parameters were monitored to assess the sample quality. The sequencing reads passing the QC parameter values were aligned to the hs38DH human reference for variant calling. The GATK MuTect2 was used to call and filter somatic variants.

### Immunocytochemistry Assay

Immunofluorescence staining was performed according to our previously published report (20). Human prostate cancer and adjacent normal prostate cells were plated onto enhanced chemiluminescence (ECL)-coated chamber slides and grown in KSFM media (Gibco). The cells were fixed in −20°C methanol, air dried, and rehydrated in 1X PBS. After blocking (0.2% BSA), cells were incubated with the primary antibodies [AR (1:500, catalog no. SC7305, Biotechnology), CK-8 (1:100, catalog no. MA5-14476, RRID:AB_10985243, Thermo Fisher Scientific/Invitrogen), CK-18 (1:100, MA5-12104, RRID:AB_10981680, Thermo Fisher Scientific/Invitrogen), HMW CK (1:100, catalog no. MA5-12135, RRID:AB_10983918, Thermo Fisher Scientific/Invitrogen), TOPK (1:10,000, catalog no. Ab226923, Abcam), c-MYC (1:500, catalog no. 5605, Cell Signaling Technology), and N-MYC (1:200, catalog no. 10159-2-AP, Protein Tech)] overnight at 4°C, followed by incubation with the Alexa Fluor 594–conjugated secondary antibodies (Molecular probes/Invitrogen) for 1 hour. Slides were washed three times with 1X PBS, counterstained with DAPI, mounted and viewed under a fluorescent Olympus BX microscope (Olympus Corp). Images were captured at the same magnification (20X) and then imported into Adobe Photoshop.

### Western Blot Analysis

The expression levels of AR and p53 were examined by Western blotting. Proteins were extracted and quantified using Nanodrop 1000 (Thermo Fisher Scientific). Equal amounts of proteins were separated in10% SDS-PAGE gel (Invitrogen) and blotted onto the polyvinylidene difluoride membrane (GE Healthcare Life Sciences). Membranes were probed with antibodies against the proteins [AR (Cell Signaling Technology, catalog no. 3202, RRID:AB_2060162) and p53 (Santa Cruz Biotechnology, catalog no. sc-126, RRID:AB_628082)] and β-actin (Sigma-Aldrich, catalog no. A3854, RRID:AB_262011) as control. Membranes were incubated with second antibodies, followed by detection of ECL reagent (GE Healthcare Life Sciences), and the Amersham Imager 600 (GE Healthcare Life Science).

### Cytotoxicity Assay

Cells were seeded for 24 hours in a 96-well plate in triplicates, followed by a 96-hour treatment with selected compounds (bicalutamide, catalog no. S1190, olaparib; catalog no. S1060, niraparib; catalog no. S7625, Selleckchem) at doses in a range of 0.25–50 μmol/L. DMSO (0.1%) treatment served as a control. To determine viability, a WST-8 [2-(2-methoxy-4-nitrophenyl)-3-(4-nitrophenyl)-5-(2,4-disulfophenyl)-2H-tetrazolium, monosodium salt] assay was performed using Cell Counting Kit-8 (SKU:CK04, Dojindo). The absorbance was measured at 450 nm after 1.5 hours of incubation. The effective concentration (EC_50_) values required to obtain a 50% response were calculated from sigmoidal dose-response curves using GraphPad Prism (RRID:SCR_002798).

### Data Availability

The data generated in this study, including derived data in support of the authentication of the cell models, are available upon request from the corresponding author. The full genomic sequence data generated in this study will be made available when the comprehensive datasets are reported in a subsequent publication by the corresponding author.

## Results

### Clinical Characterizations

Patients enrolled into IRB study number 2017-0614 underwent preoperative physical examination and biopsy to establish a cancer diagnosis. Prostatectomies were performed using the DaVinci robot. Postoperative histologic pathologies were compared with the preoperative values. Approximately 20% disagreements of patients’ Gleason grades were observed on comparison of preoperative to postoperative diagnoses. The specimens were annotated with age, race, stage, PSA, and postoperative Gleason grade as summarized in Supplementary Table S1.

### Establishment of Primary Prostate Tumor and Non-tumor Cultures Derived from the Same Patient

Localized prostate cancer is morphologically heterogeneous with multiple cell types (21). To inhibit the growth of nonepithelial cells, KSFM was used for primary cell outgrowth from tissues to expand prostate epithelial cells. The cultures were expanded up to four passages and tended to senesce after that.

To further characterize the AA cells, we selected 10 primary cell cultures for authentication by STR and karyotype analyses for this study. STR defined the cultures as unique. As an example, Supplementary Fig. S1A summarizes results obtained from analyses of 15 autosomal STR loci and the gender identification locus amelogenin shown as a male. The profile of cell culture AP4T at passage 1 did not generate a match when compared against the ATCC, DSM2, and Cellosaurus human cell STR profile databases. These data show that AP4T has a unique profile with no mouse DNA to contaminate the specimen.

For karyotyping, 20 metaphase cells were analyzed from 10 tumor and non–tumor-derived cell cultures. All karyotypes were male and showed no gross abnormalities, within the limits of resolution of this standard cytogenetic methodology (Supplementary Fig. S1B). The method does not detect subtle rearrangements, low-level mosaicism or microdeletions.

To expand the primary cell cultures beyond to the passage limit before senescing, cells were grown in CR culture conditions (Supplementary Fig. S1C). The CR growth conditions permit culture expansion to the large numbers of cells (∼10^8^) for both tumor- and normal-derived cultures.

We observed distinct cell morphologies on comparison of normal- and tumor-derived cell cultures. Normal cells (N) grow in flat, fan-like and sheet-like structures (lamellipodia) compared with that of tumor-derived cells (compact epithelial growth). Under the CRC, J2 fibroblasts were displaced by epithelial cells that formed colonies and expanded by day 7 after plating. These cells are easily separated by sequential trypsin treatment (0.05% for fibroblasts and 0.25% for epithelial cells).

### Cellular Annotation

**FIGURE 1:**
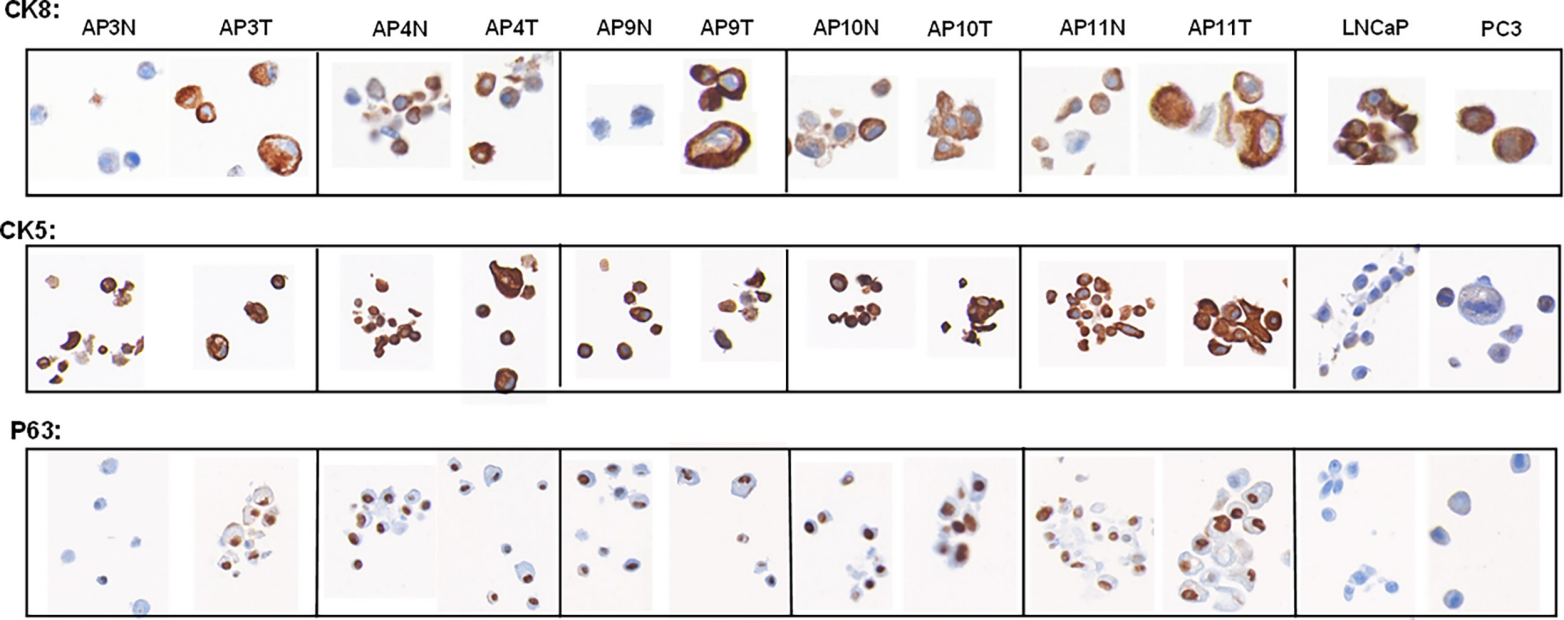
Immunostaining of paraffin-embedded cells expressing markers CK8, CK5, and p63. Normal and cancer cells were plated for 48 to 72 hours and stained with basal and luminal markers (AR, CK8, and p63). The whole slide was scanned with the Vectra system. PC3 and LNCaP cells served as controls. AR and p53 were undetectable or weak, respectively (data not shown). Magnification 20×.

Human prostate cancers have complex cellular compositions, expressing distinct cytokeratin subtypes that have been correlated to clinical prognosis and the survival (22). Using paraffin-embedded cell-based microarrays (CMA), we examined cellular phenotypes by immunostaining. The selected cell cultures were examined for expression of basal and luminal phenotypes. LNCaP (AR-dependent) and PC3 (AR-independent) cells served as controls for AR and CK8 expression. Immunostaining revealed that the majority of normal- and tumor-derived cells expressed both luminal (CK8) and basal (CK5, p63) markers (Fig. 1). Most tumor cells contained heterogeneous populations with variable morphologies and large cells, some with multiple nuclei. Normal cells derived from patients (AP3N and AP9N) were negative for CK8 but AP4N, AP10N, and AP11N were positive for CK8 with variable intensity. Although CK8 was observed in all tested tumor cells, CK5 and p63 were also observed, suggesting these cells have a bimodal and complex cellular phenotype. Such observations are consistent with reported findings that tumors expressing combined luminal and basal phenotypes are correlated with the poor prognosis and histologic grade (22). Expression of the basal markers has also been reported in invasive breast carcinoma (23). Our data demonstrate differential expression levels of both basal and luminal markers in these cells, interpreted as heterogeneity in localized prostate cancer.

**FIGURE 2:**
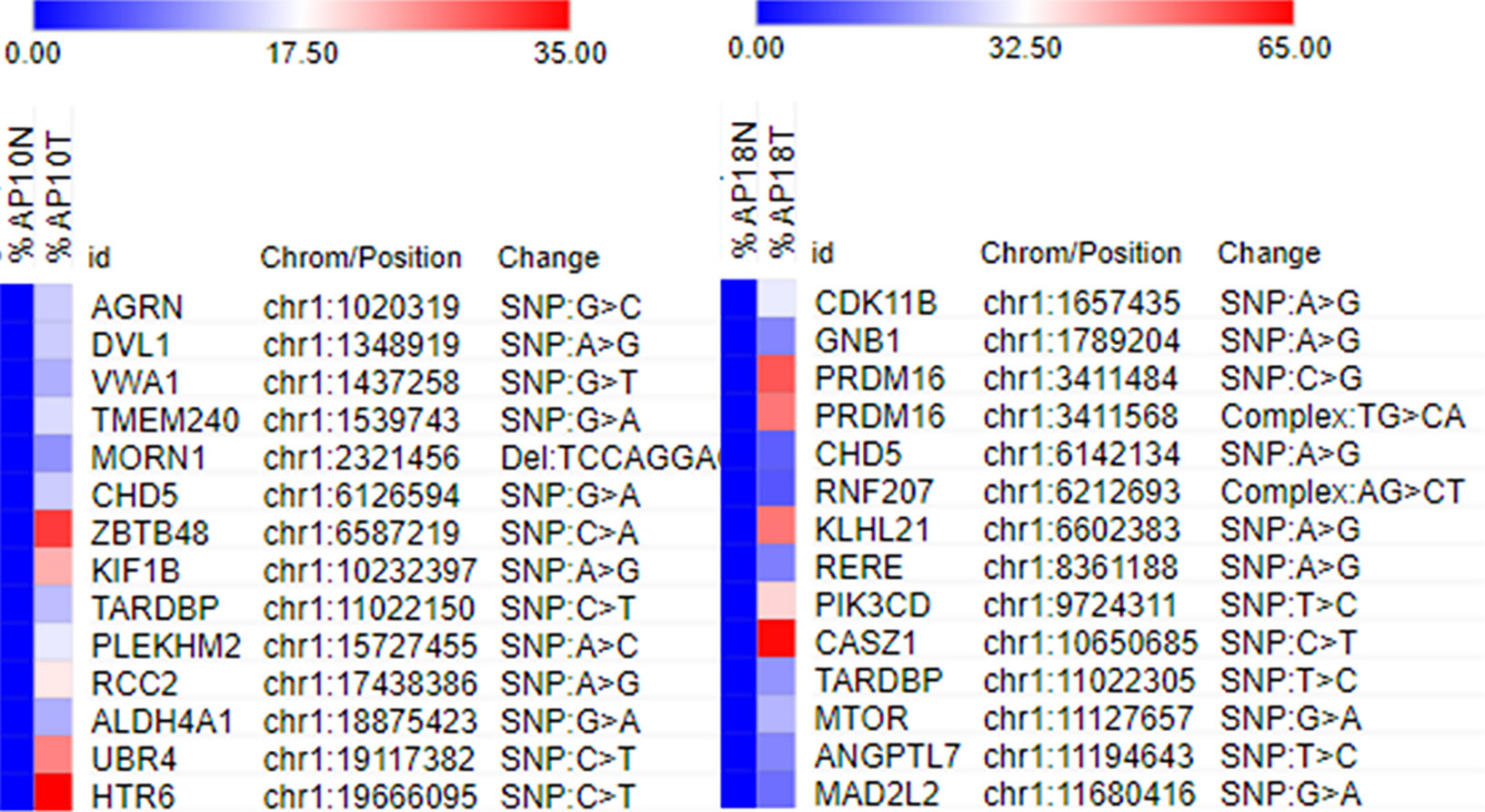
The heatmap depicts variant allele fraction unique to AP10 and AP18 tumor-derived cells. A panel of the genetic profiling presents a number of somatic variants that are unique to the tumor-derived cells, matching the normal-derived to tumor-derived cell culture with a somatic panel of SNPs. The percentage of sequencing reads that match the hs38DH human reference were used for somatic variant calling using the GATK MuTect2 and visualized using Morpheus from the Broad Institute. “0” is the reference and a nonzero value is that alternate in the list. A relative color scheme presents with the minimum and maximum values (%) in each row to convert values to colors.

To further characterize these patient-derived primary tumor cells, WGS was performed using the paired normal- and tumor-derived cell cultures. The preliminary genomic profiles of selected groups demonstrated matching the normal-derived to tumor-derived cell culture with somatic variants that are unique to the tumor cells (Fig. 2), supporting that tumor cells are tumor derived. The comprehensive genomic profiling will be released elsewhere.

### Molecular and Cellular Characterizations

Expression of AR and p53 proteins was further examined by Western blot analysis (Supplementary Fig. S2). AR was highly expressed in LNCaP cells and not expressed in PC3, serving as controls. The AA prostate expressed p53 at variable levels. AR expression was not observed in these prostate-derived cells. Cells derived from two tumor locations in separate regions of the prostate, AA4 and AA11 patients (LLA; low left anterior and RM; right middle) showed similar expression levels of p53 but no AR. p53 expression was low in both tumor-derived cells.

**FIGURE 3:**
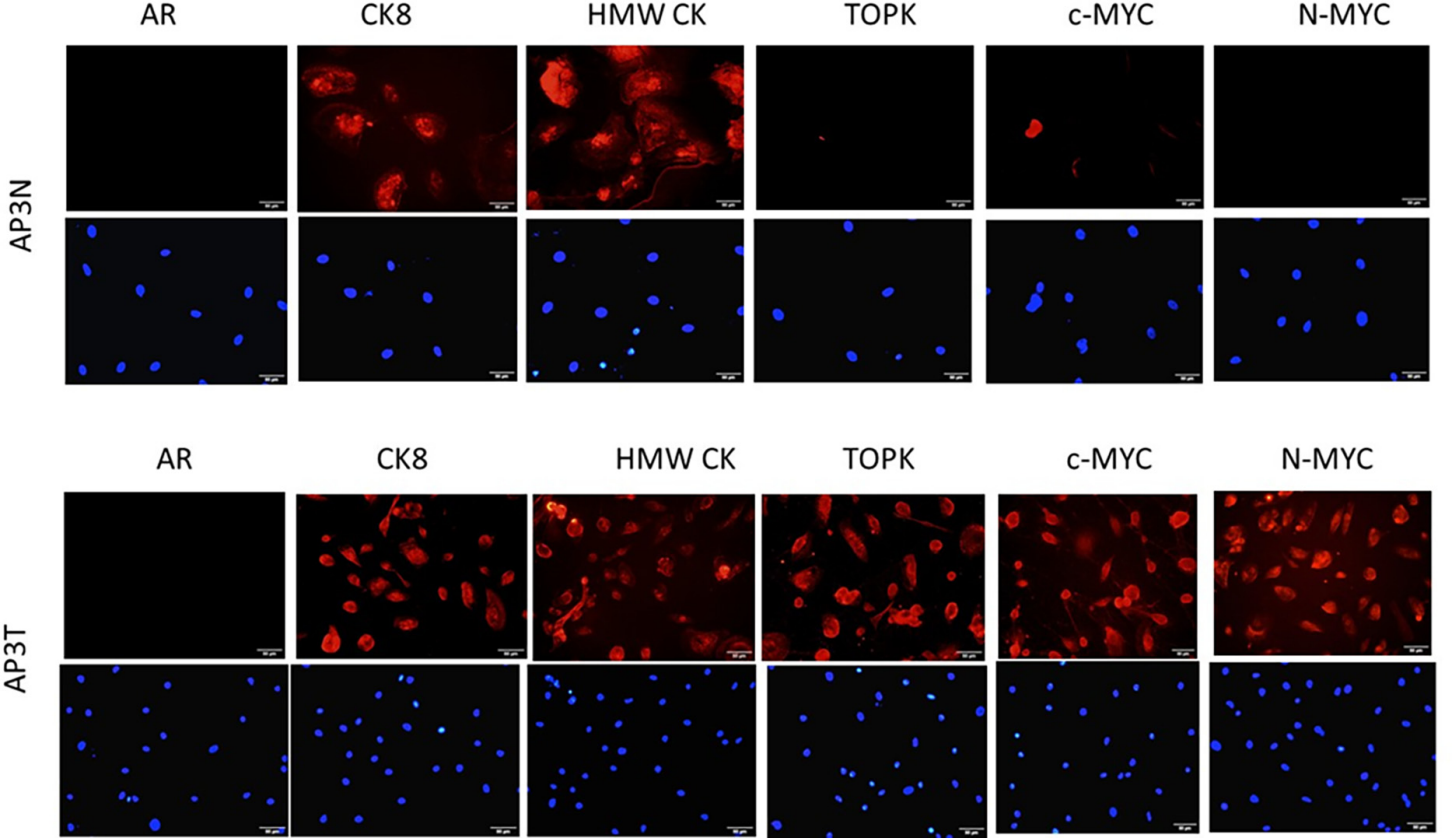
Immunocytochemistry analysis of AP3 normal (N)- and tumor (T)-derived paired cell cultures. Normal and tumor cells from AP3 were stained with indicated antibodies. DAPI (bottom in blue) stained the nucleus. CK8, HMW CK, and TOPK were mainly located in the cytoplasm while c-MYC and N-MYC were in the nucleus. Additional immunostaining data in Supplementary Fig. S3.

Further cellular characterizations were performed by examining relevant protein expression levels. Immunostaining for AR, CK8, high-molecular weight keratin (HMWCK), TOPK, c-MYC, and N-MYC demonstrated differential levels of CK8 and N-MYC in AP3 cancer and normal cells with HMWCK as a basal marker. AP3N and AP3T cells are shown as representative images in Fig. 3, and AP4N/T and AP10N/T images in Supplementary Fig. S3. Differential expression levels of basal and luminal markers were observed in normal compared with tumor cells (Table 1). Although both AR and CK8 are known as luminal markers, no AR expressions were noted. Abundant CK8 staining was present in both normal and cancer cells. As target molecules with critical roles in various cancers, TOPK, c-MYC, and N-MYC were exclusively expressed only in tumor cells. Consistent with studies demonstrating upregulation of c-MYC and N-MYC (21, 24) as well as TOPK in prostate cancer (25), these molecules were expressed at high levels. c-MYC and N-MYC were amplified mainly in the nucleus and TOPK, which has been correlated with metastasis and poor prognosis, was in the cytoplasm (25). Table 1 summarizes the clinical characteristics and immunocytochemistry analyses of patient-derived normal and cancer cells examined.

**TABLE 1:** Summary of clinical characterization and cellular markers

Clinical characterization					Cellular annotations						
ID	Age	Gleason	PSA (ng/mL)	T stage	Cells	AR	CK8	HMW CK	c-Myc	N-Myc	TOPK
AP3	57	4+3 = 7	19.9	T2	normal	N: −	Y: +++	Y: +++	N: −	N: −	N: −
					tumor	N: −	Y: +++	Y: +++	Y: +++	Y: +++	Y: +++
AP4	65	3+3 = 6	5.6	T2	normal	N: −	Y: +++	Y: +++	N: −	N: −	N: −
					tumor	N: −	Y: +++	Y: +++	N: −	N: −	Y: +++
AP10	54	3+4 = 7	3.4	T2	normal	Y: +++	Y: +++	ND	Y: +	N: −	ND
					tumor	N: −	Y: +++	ND	Y: +++	Y: +++	ND
AP11	69	4+4 = 8	9.23	T2	normal	N: −	Y: +++	Y: +++	N: −	N: −	ND
					tumor	N: −	Y: +++	Y: +++	Y: +++	Y: +++	ND
AP15	68	4+5 = 9	9	T2	normal	N: −	Y: +++	Y: +++	N: −	N: −	ND
					tumor	N: −	Y: +++	Y: +++	Y: +++	Y: +	ND
AP17	55	3+3 = 6	13.1		normal	N: −	Y: +++	Y: +	N: −	N: −	ND
					tumor	N: −	Y: +?	Y: +	Y: +++	Y: +++	ND
AP18	63	4+3 = 7	6.4	T2	normal	N: −	Y: +++	Y: +++	N: −	N: −	ND
					tumor	N: −	Y: +++	Y: +++	N: −	N: −	ND

### Efficacy Screening of Therapeutic Compounds on Cell Survivals

**FIGURE 4:**
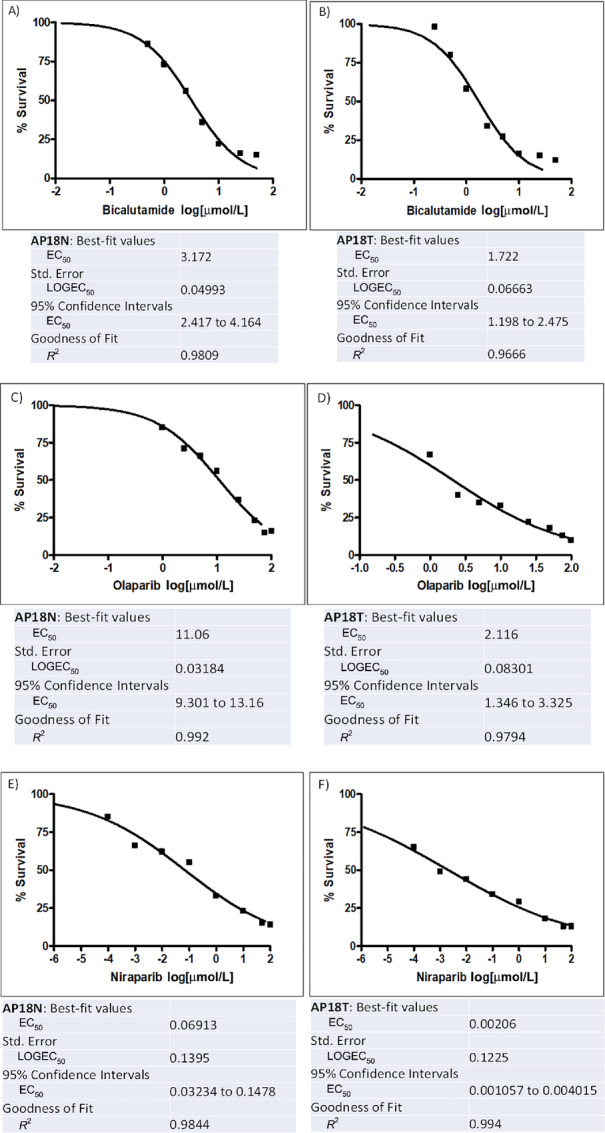
Cytotoxicity assays: AP18 normal and tumor (AP18T) cells were treated for 96 hours with indicated drugs; normal (**A**) and tumor (**B**) with bicalutamide, normal (**C**) and tumor (**D**) with olaparib, normal (**E**) and tumor (**F**) with niraparib at doses in a range of 0.25–50 μmol/L. DMSO (0.1%) served as a control. The EC_50_ values were calculated from sigmoidal dose–response curves using Prism.

We tested the feasibility of using these cell cultures in testing for responses to therapeutic agents, including bicalutamide (antiandrogen) used to treat metastatic prostate cancer and two PARP inhibitors (olaparib and niraparib) used to treat castrate-resistant or metastatic prostate cancer (Fig. 4). The data demonstrated that bicalutamide was less cytotoxic to normal cells (AP18N: EC_50_ at 3.2 μmol/L) compared with tumor cells (AP18T: EC_50_ at 1.7 μmol/L). In addition, both PARP inhibitors were less toxic to normal cells than to tumor cells. However, normal and tumor cells were extremely sensitive to niraparib (EC_50_ at 0.07 μmol/L in AP18N and EC_50_ at 0.0021 μmol/L in AP18T) as compared with olaparib (EC_50_ at 3.043 μmol/L in AP18N; EC_50_ at 2.172 μmol/L in AP18T). Overall, the data demonstrated differential cellular survivals, supporting these cells as a potential cellular model for cell-based drug testing.

## Discussion

AA men diagnosed with prostate cancer have early onset disease, often display aggressive disease progression and experience worse outcomes than men of other racial and/or ethnic groups. We have established human normal and tumor cell cultures from prostatectomy specimens obtained from AA patients for use in genomic and cellular studies of factors that may underlie these health care disparities.

Prostate tumor heterogeneity has been attributed to underlying tumor aggressiveness and clinical outcomes. Human prostate cells contain three types of terminally differentiated epithelial cells, including basal, luminal, and neuroendocrine (26). Engineered model systems have been developed to study the genetics and biology of prostate cancer (reviewed in ref. 24). To develop cell-based models, we used conditional reprogramming technology for expanding paired cell culture strains grown from biopsy material from normal prostate regions and from prostate tumors, derived from the same patient. These cells are able to undergo more divisions than can be achieved in conventional tissue culture. The phenotypes of cell cultures grown under CRC technology have been attributed to a “reprogrammed stem-like” state of epithelial cells, in which the cells maintain their original karyotypes, resulting in the loss of some differentiation markers. Removal of CRC conditions restores the capacity for cell differentiation. Normal CR cell cultures retain a normal karyotype and differentiation potential, and CR cells derived from tumors retain their tumorigenic phenotypes (11–16).

Traditionally, prostate adenocarcinoma has been characterized by the absence of a basal layer. The expression of cytokeratin subtypes has been assessed to determine distinct phenotypes relevant to histologic grade and clinical prognosis. However, growing evidence supports the concept that a subset of basal cells can play a role in prostate carcinogenesis (26, 27). Our immunochemical data demonstrate that the patient-derived tumor cells have a bimodal and complex cellular phenotype by expression of basal and luminal markers. CK8 was observed in all tumor cells but they are also CK5 and p63 positive. Such observations have been previously reported by others (27). Both basal and luminal cells can serve as cells of origin for prostate cancer (28) and that 66% of formalin-fixed, paraffin-embedded prostatectomy samples (*n* = 1,567) can be classified as luminal and 33% as basal subtypes (22), support divergent clinical behavior. Furthermore, tumors expressing combined luminal- and basal-like phenotypes have correlated with poor prognosis and histologic grade. The basal marker (CK5) expression was significant in invasive breast carcinoma associated with poor prognostic features and had an independent prognostic impact in patients without metastasis (23). Tumors expressing basal or combined basal and luminal markers were more often stage III tumors with poor outcome. Such a phenotype was identified in 62% of poorly differentiated breast cancers and tended to have more frequent metastasis (29). Our preliminary genomic profiles of selected groups demonstrated matching the normal-derived to tumor-derived cell culture with somatic variants that are unique to the tumor cells.

Prior studies have demonstrated an increase of MYC in more than 50% of prostate cancer tumors (30–32). Overexpression of MYC in the prostate results in prostatic intraepithelial neoplasia (PIN) with progression to invasive adenocarcinoma (31). Recent studies have also demonstrated the role of TOPK, a MAPKK-like kinase, in prostate cancer (25, 33). In AA prostate cancer cells examined here, differential expression of MYC and TOPK was observed in tumor-derived cultures as compared with cells derived from normal tissues in the prostate. These proteins are known as oncogenic and correlate with metastasis and poor clinical prognosis.

The cytotoxicity data reveal differential sensitivities of normal- and tumor-derived prostate epithelial cells following exposure to an AR antagonist and to two PARP inhibitors. These drugs conferred higher cytotoxicity against tumor cells than against normal cells, suggesting a therapeutic window. Considering the feasibility of establishing cell cultures from core needle biopsies (data not shown), this system has capability for cell-based drug testing for precision medicine.

To understand prostate cancer complexity and identify a key determinant of cell heterogeneity, most large-scale sequencing studies have used microdissected paraffin-embedded tissues containing heterogeneous types of cells, including epithelial and nonepithelial cells, for DNA or RNA resources (34). Although future studies will characterize cellular phenotypes with molecular and genetic markers, the current study demonstrates differences in cellular phenotypes, expressed molecules, and drug responses between normal- and cancer-derived tumor cells. The use of these cells also enables analysis of the genomic mutational landscape. The CRC expanded cells offer a more homogeneous prostate epithelial cell population for genomic testing, which is currently under investigation. Further genetic and cellular characterization of these established cell cultures should improve our understanding of underlying mechanisms in health disparities of AA patients presenting with prostate cancer. Identifying genetic alterations associated with the aggressive nature of these tumor cells will address underlying molecular mechanisms.

To our knowledge, our cellular models are the only known matched sets of normal and tumor cells derived from AA patients. These cell models allow clinical characterization with integration of disease phenotype and impact of lifestyle to support basic science and population science research into AA health disparities.

## Supplementary Material

Supplementary Table ST1. Table S1. Clinical characterization of prostate cancer patients.

Supplementary Figure SF1 Figure S1: A) Results obtained from analyses of 15 autosomal short tandem repeat (STR) loci identify the male locus amelogenin. The profile of L/P4T is unique. No mouse DNA was detected for this sample. B) Karyotype of AP4T. Of 20 metaphases counted, 5 metaphases were analyzed. 3 metaphases were then karyotyped at 425 banding resolution. The image shows the 46,XY, male karyotype. Normal karyotype (male) within the limits of resolution attained. C) Cell growth and morphology under CR condition. Non-cancerous and cancerous cells derived from AP4 were expanded using CR condition. The images of 10x and 20x are from passage 1 and stained with H&E. The bottom image shows clonogenic growth of epithelial cells and J2 fibroblasts after 4 days and 7 days of culture. On day 7, clonogenic epithelial cells are marked in yellow arrow and J2 fibroblasts in cyan blue arrow.

Supplementary Figure SF2. Figure S2. Western analysis of AR and p53. Cells were examined for expression levels of AR and p53 in a set of cell cultures. LNCaP (AR-dependent and p53+) and PC3 (AR-independent and p53 null) cells were used as controls for AR and p53 expression. Actin was used as the loading control.

Supplementary Figure SF3. Figure S3: Immunocytochemistry analysis of normal (N) and tumor (T)- derived paired cell cultures of AP4 and AP10. Normal and tumor cells from AP4 and AP10 were stained with indicated antibodies. DAPI (bottom in blue) stained the nucleus. CK8, HMW CK, and TOPK were mainly located in the cytoplasm.

Supplementary Protocol SP1 Material and methods of cell culture and CRC protocol
