# Supplementary material for: Novel Paired Normal Prostate and Prostate Cancer Model Cell Systems Derived from African American Patients
Source: Cancer Res Commun. 2022 Dec 13;2(12):1617–25. doi: 10.1158/2767-9764.CRC-22-0203 (PMC10035501; doi:10.1158/2767-9764.CRC-22-0203)
Supplement: Supplementary Table ST1. — Table S1. Clinical characterization of prostate cancer patients. [file crc-22-0203-s01.pdf]

Table S1. Clinical characterization of prostrate cancer patients

Patient	Age	PSA (ng/ml)	Gleason (3+3)	Gleason (3+4)	Gleason (4+3)	Gleason (≥4+4)	T stage (pathologic)
AA (n=4)	≤50 (n=4)	3.6 – 8.5	1	3	0	0	T2-T3
AA (n=12)<br>CA (n=6)	51-59 (n=18)	1.5-19.9	5	8	1	4	T2-T3
AA (n=21)<br>CA (n=4)<br>AS, H (n=2)	60-69 (n=27)	0.3-18.8	4	10	4	9	T2-T3
AA (n=5)<br>CA (n=4)	≥70 (n=9)	1.1 - 30.8	0	5	0	4	T2-T3

42 African American (AA); 14 Caucasian American (CA); 2 Asian and Hispanic (AS&H)
