# Supplementary material for: Novel Paired Normal Prostate and Prostate Cancer Model Cell Systems Derived from African American Patients
Source: Cancer Res Commun. 2022 Dec 13;2(12):1617–25. doi: 10.1158/2767-9764.CRC-22-0203 (PMC10035501; doi:10.1158/2767-9764.CRC-22-0203)
Supplement: Supplementary Figure SF1 — Figure S1: A) Results obtained from analyses of 15 autosomal short tandem repeat (STR) loci identify the male locus amelogenin. The profile of L/P4T is unique. No mouse DNA was detected for this sample. B) Karyotype of AP4T. Of 20 metaphases counted, 5 metaphases were analyzed. 3 metaphases were then karyotyped at 425 banding resolution. The image shows the 46,XY, male karyotype. Normal karyotype (male) within the limits of resolution attained. C) Cell growth and morphology under CR condition. Non-cancerous and cancerous cells derived from AP4 were expanded using CR condition. The images of 10x and 20x are from passage 1 and stained with H&E. The bottom image shows clonogenic growth of epithelial cells and J2 fibroblasts after 4 days and 7 days of culture. On day 7, clonogenic epithelial cells are marked in yellow arrow and J2 fibroblasts in cyan blue arrow. [file crc-22-0203-s02.pdf]

Figure S1:

A) Short Tandem Repeats (STR) profiling of AP4T.

DNA Analysis									
	D3S1358	D7S820	vWA	FGA	D8S1179	D21S11	D18S51	D5S818	D13S317
SMP1	14, 18	9, 10	13, 16	20, 23	14	31, 35	12, 17	11, 13	12
REF	NA	NA	NA	NA	NA	NA	NA	NA	NA
	D16S539	TH01	TPOX	CSF1PO	AMEL	Penta D	Penta E		Mouse
SMP1	12, 13	7	10, 11	11	X, Y	11, 12	9, 20		NA
REF	NA	NA	NA	NA	NA	NA	NA		NA

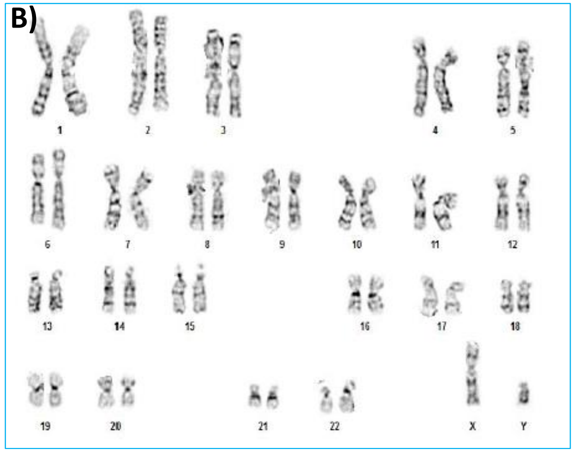

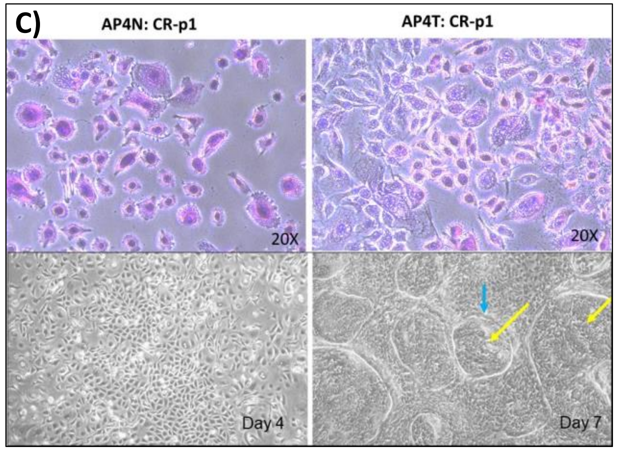

Figure S1: A) Results obtained from analyses of 15 autosomal short tandem repeat (STR) loci identify the male locus amelogenin. The profile of L/P4T is unique. No mouse DNA was detected for this sample. B) Karyotype of AP4T. Of 20 metaphases counted, 5 metaphases were analyzed. 3 metaphases were then karyotyped at 425 banding resolution. The image shows the 46,XY, male karyotype. Normal karyotype (male) within the limits of resolution attained. C) Cell growth and morphology under CR condition. Non-cancerous and cancerous cells derived from AP4 were expanded using CR condition. The images of 10x and 20x are from passage 1 and stained with H&E. The bottom image shows clonogenic growth of epithelial cells and J2 fibroblasts after 4 days and 7 days of culture. On day 7, clonogenic epithelial cells are marked in yellow arrow and J2 fibroblasts in cyan blue arrow.
