# Supplementary material for: Novel Paired Normal Prostate and Prostate Cancer Model Cell Systems Derived from African American Patients
Source: Cancer Res Commun. 2022 Dec 13;2(12):1617–25. doi: 10.1158/2767-9764.CRC-22-0203 (PMC10035501; doi:10.1158/2767-9764.CRC-22-0203)
Supplement: Supplementary Figure SF2. — Figure S2. Western analysis of AR and p53. Cells were examined for expression levels of AR and p53 in a set of cell cultures. LNCaP (AR-dependent and p53+) and PC3 (AR-independent and p53 null) cells were used as controls for AR and p53 expression. Actin was used as the loading control. [file crc-22-0203-s03.pdf]

Supplementary Figure S2.

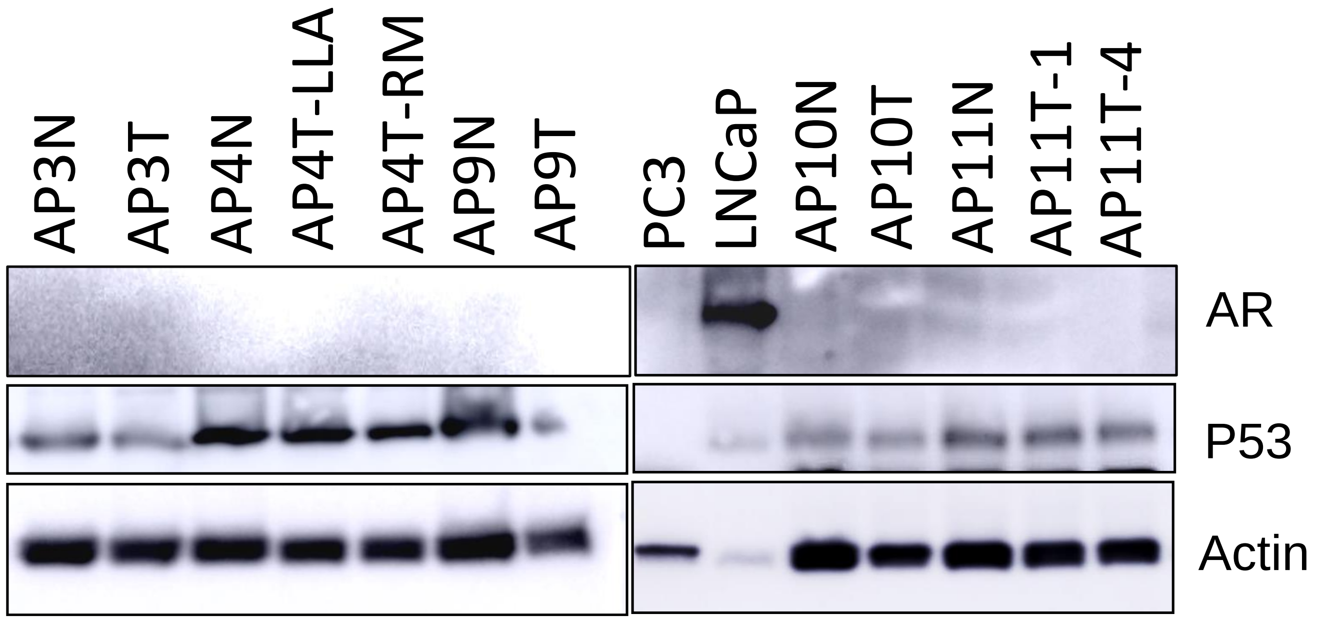

Figure S2. Western analysis of AR and p53. Cells were examined for expression levels of AR and p53 in a set of cell cultures. LNCaP (AR-dependent and p53+) and PC3 (AR-independent and p53 null) cells were used as controls for AR and p53 expression. Actin was used as the loading control.
