# Supplementary material for: Novel Paired Normal Prostate and Prostate Cancer Model Cell Systems Derived from African American Patients
Source: Cancer Res Commun. 2022 Dec 13;2(12):1617–25. doi: 10.1158/2767-9764.CRC-22-0203 (PMC10035501; doi:10.1158/2767-9764.CRC-22-0203)
Supplement: Supplementary Figure SF3. — Figure S3: Immunocytochemistry analysis of normal (N) and tumor (T)- derived paired cell cultures of AP4 and AP10. Normal and tumor cells from AP4 and AP10 were stained with indicated antibodies. DAPI (bottom in blue) stained the nucleus. CK8, HMW CK, and TOPK were mainly located in the cytoplasm. [file crc-22-0203-s04.pdf]

Supplementary Figure S3.

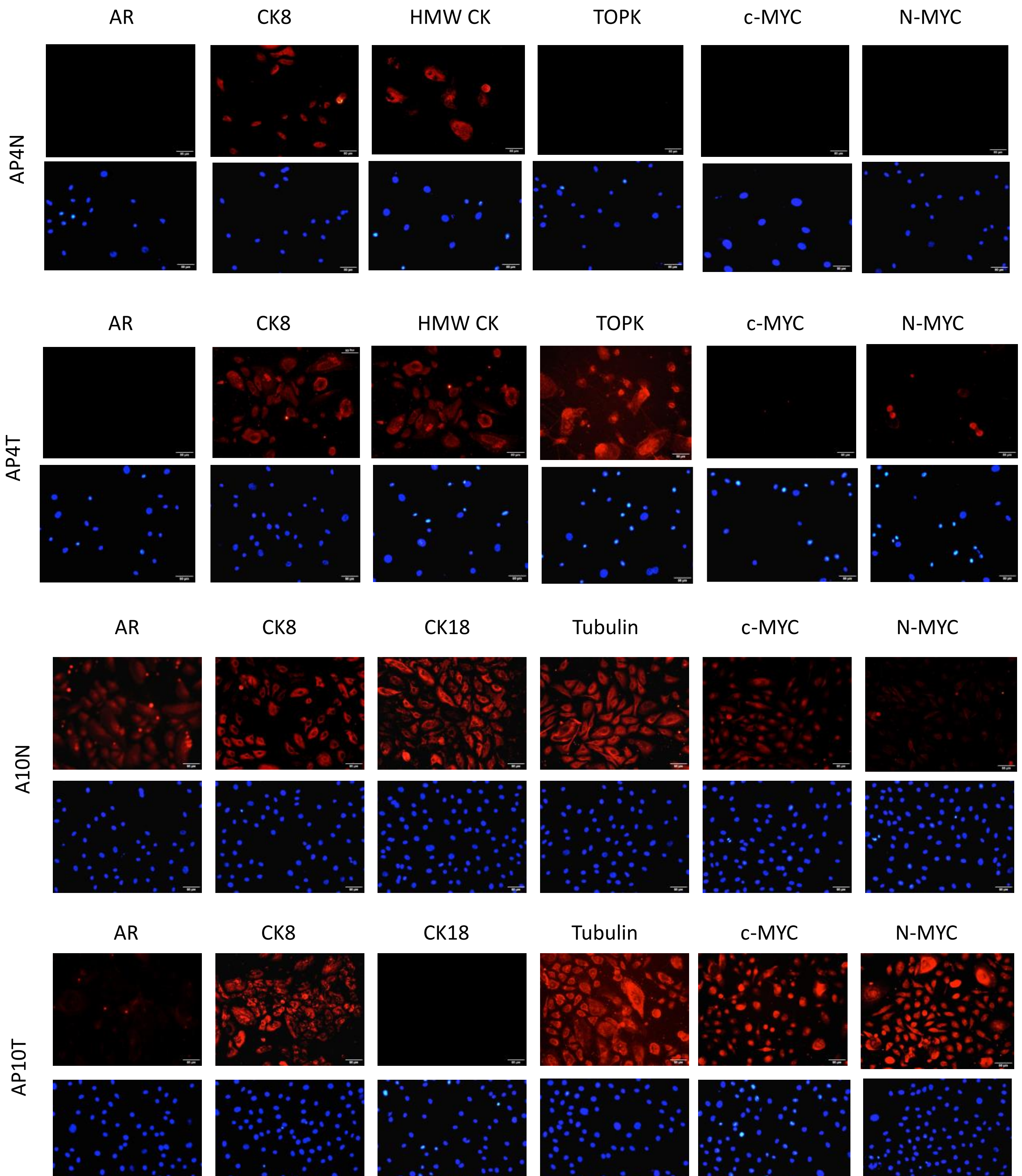

Figure S3: Immunocytochemistry analysis of normal (N) and tumor (T)- derived paired cell cultures of AP4 and AP10. Normal and tumor cells from AP4 and AP10 were stained with indicated antibodies. DAPI (bottom in blue) stained the nucleus. CK8, HMW CK, and TOPK were mainly located in the cytoplasm.
