# Supplementary material for: Novel Paired Normal Prostate and Prostate Cancer Model Cell Systems Derived from African American Patients
Source: Cancer Res Commun. 2022 Dec 13;2(12):1617–25. doi: 10.1158/2767-9764.CRC-22-0203 (PMC10035501; doi:10.1158/2767-9764.CRC-22-0203)
Supplement: Supplementary Protocol SP1 — Material and methods of cell culture and CRC protocol [file crc-22-0203-s05.pdf]

Primary Prostate Cell Culture from prostatectomies

COMMENTS 0

RESERVED DOI:
10.17504/protocols.io.5qpvornyzv4o/v1

Mira Jung[1]

[1]Georgetown University Medical Center

jungm

WORKS FOR ME 1

ABSTRACT

Based on the tissue collection protocol under the Georgetown University's Institutional Review Board approval, fresh clinical specimens were transferred from the operating room to surgical pathology, processed and examined by the pathologist for areas of tumor or normal (non-tumor) regions for biopsies. The specimens were placed on ice and transferred to the laboratory for processing, outgrowth and expansion. To generate primary cell cultures, the minced tissues were cultured in collagen-coated cell culture dishes with keratinocyte serum-free medium (K-SFM) supplemented with human recombinant epidermal growth factor (rEGF) and bovine pituitary extract (BPE) at the time of use. Tissue explants were grown to confluence, and aliquots of the primary cultures were frozen and stored in liquid nitrogen. To further expand primary cells, irradiated 3T3 J-2 cells derived from mouse embryonic fibroblast cells were used as feeder cells for growing epithelial cells. J2 cells were cultured in complete DMEM, 10% heat inactivated FBS and 1 % Pen Strep. The complete DMEM and Ham's F-12 nutrient mix (25%) supplemented with hydrocortisone, epidermal growth factor, bovine insulin, cholera toxin, amphotericin B, gentamicin, and Rock Inhibitor (Y-27632) was used to grow primary epithelial cells with irradiated J2 cells with 30 Gy of gamma radiation.

PROTOCOL INFO

Mira Jung . Primary Prostate Cell Culture from prostatectomies. **protocols.io**
https://protocols.io/view/primary-prostate-cell-culture-from-prostatectomies-cgd4ts8w

CREATED

Sep 09, 2022

LAST MODIFIED

Sep 12, 2022

PROTOCOL INTEGER ID

69788

MATERIALS TEXT

Biopsy collection medium:
Keratinocyte serum-free medium (K-SFM) (Gibco, Invitrogen, Corp. Cat. No. 17005-042) plus 2.5 mg/ml epidermal growth factor (EGF) human recombinant (Cat. No. 10450-013) and 25 mg/ml bovine pituitary extract (BPE) (Cat. No. 13028-014) with5% heat-inactivated fetal bovine serum (FBS) (Gibco, Invitrogen, Corp.), 1% PSN (Penicillin 5 mg/Streptomycin 5 mg/ml; Gibco, Invitrogen, Corp.), 1% fungizon (Amphotericin B 250 mg/ml, Gibco, Invitrogen, Corp. Cat. No. 15290-034), and 10 mM HEPES (Sigma Cat. No. H4034).

Supplies:

- Type-1 collagen-coated petri dishes (Becton-Dickinson, Boston, MA) 100 cm.
- Scalpel blades.
- Sterile petri dishes (100 cm) for dissection steps.
- Incubator at 37°C and 5% CO_2.

Expansion of primary cells under conditional reprograming cell culture (CRC):

Complete DMEM: 500 mL DMEM (100mg/mL glutamine, 4.5 g/L D-Glucose) plus 50 mL Heat inactivated FBS (heated in 60°C water bath for 20min) and 5 mL PEN/STREP
F-Medium:375 mL Complete DMEM and 125 mL Ham’s F-12 nutrient mix supplementedwith25ng/mL Hydrocortisone with 0.125 ng/mL EGF, 5 mg/mL of bovine insulin, 0.1nmol/L cholera toxin, amphotericin B, gentamicin, and 10 mM Rock Inhibitor (Y-27632).

Isolation of primary cells from human prostate tissue

1 Working under a biosafety cabinet, transfer the fresh tissue into the plate.

2 With a sterile blade, make a fine mince of the tissues.

3 Add 10 ml of collection medium, mix well, and transfer into collagen-coated dishes.

4 Add 5 ml of collection media and place dishes into a 37°C 5% CO 2 incubator.

5 Within a week the minced tissue should be completely attached to the bottom surface of the culture dishes.

6 Do not change the medium before 1 week or the cells may not have a chance to adhere and grow.

7 After 7 days of culture, aseptically remove the collection medium and add 10 ml of keratinocyte growth medium (KGM) (CC-4152, Lonza) with no 5%FBS into a petri dish.

8 Incubate the cells at 37° C in humidified air of 5% CO 2 until reaching semiconfluency (about 70–80%), changing the medium twice weekly.

9 Freeze aliquots of primary cells using 10% DMSO in KGM.

Expansion of primary cells under conditional reprograming cell cultu

10 Steps:

11 Grow J2 feeder cells with 25 mL Complete DMEM to 75-80% confluency (1-1.5 x 107 cells) in T-175 flask.

12 Remove media and trypsinize cells using 10 mL of 0.25% Trypsin for minimum of 2 min.

13 Make sure cells are floating as single cells and not in clumps or clusters by pipetting several times or vortexing cell suspension and verify by microscope.

14 Neutralize Trypsin with 10 mL of Complete DMEM.

15 Transfer cell suspension into a conical tube and spin down cells at 3000 rpm for 5 min.

16 Aspirate out the supernatant.

17 Resuspend cells with 10 mL Complete DMEM.

18 Irradiate cell suspension with a Cesium Irradiator at 30 Grey.

19 Spin down cells and aspirate out the supernatant.

20 Resuspend cells with fresh 10 mL F-Medium (less than 2 weeks old or refresh older F-Medium by adding fresh Rock Inhibitor (10 µM Y-27632).

21 Plate irradiated J2 cells in a T25 flask (1 × 104 cells/cm2) together with epithelial cells in complete F medium.

22 Maintain cocultures at 37 °C, 95% humidity and 5% CO2 until confluent.

23 Passage cocultured cells using differential trypsin treatment.

24 Rinse coculture with PBS and incubate with 1 ml of 0.05% trypsin/EDTA solution (for a T25 flask) at room temperature for 20 s.

25 Monitor closely using a phase-contrast microscope. When the feeder cells become round and begin to detach from the substrate, tap the flask gently and remove the detached cells by aspiration.

26 Colonies of epithelial cells should remain tightly adherent. Do not extend trypsin/EDTA incubation time longer than 1 min.

27 Rinse the epithelial cells with PBS and re-treat them with 1 ml of 0.05% trypsin/EDTA at 37 °C for 5 min.

28 Stop the trypsin/EDTA incubation by adding four volumes of complete DMEM to the cells and transfer the mixture to a 15-ml centrifuge tube.

29 Centrifuge the cells at 300g for5 min at 4 °C.

30 After centrifugation, resuspend the cell pellet in complete F medium.
